# Effect of glycopyrrolate on vasopressor requirements for non-elective cesarean section under spinal anesthesia: a randomized, double-blind, placebo-controlled trial

**DOI:** 10.1186/s12871-022-01882-4

**Published:** 2022-10-25

**Authors:** Rajesh Deshar, Asish Subedi, Krishna Pokharel, Birendra Prasad Sah, Jagat Narayan Prasad

**Affiliations:** 1 Department of Anesthesiology & Critical Care Medicine, Bheri Hospital, Nepalgunj, Nepal; 2 Department of Anesthesiology & Critical Care Medicine, BP Koirala Institute of Health Sciences, Dharan, Nepal

**Keywords:** Cesarean section, Glycopyrrolate, Hypotension, Spinal anesthesia, Vasopressor

## Abstract

**Background:**

The study aimed to investigate whether prophylactic use of glycopyrrolate decreases the vasopressor requirements to prevent hypotension following spinal anesthesia during non-elective cesarean section.

**Method:**

In this double-blind randomized clinical trial, 258 patients undergoing non-elective cesarean section were randomly assigned (1:1) to receive intravenous 0.2 mg glycopyrrolate or normal saline (placebo) before spinal anesthesia. The primary outcome was phenylephrine equivalent needed intraoperatively. Secondary outcomes included incidences of maternal hypotension, reactive hypertension, bradycardia, need for atropine, tachycardia, intraoperative nausea/vomiting, shivering, pruritus, dry mouth, dizziness; neonatal APGAR score at 1 min and 5 min, neonatal resuscitation needed, NICU admission and neonatal death.

**Results:**

Three patients withdrew from the study due to failed spinal anesthesia. 128 patients in the glycopyrrolate group and 127 patients in the placebo group were analyzed. The mean phenylephrine equivalent needed was 1108.96 μg in the glycopyrrolate group and 1103.64 μg in the placebo group (mean difference, 5.32 μg [95% CI − 67.97 to 78.62]; *P* = 0.88). Hypotension occurred in 38 patients (30%) in the glycopyrrolate group as compared with 49 patients (39%) in the placebo group (*P* = 0.13). Tachycardia was reported in 70% of the participants in the glycopyrrolate group and 57% of those in the placebo group (*P* = 0.04). No statistically significant difference was noted in hypotensive episodes > 1, reactive hypertension, bradycardia, need for atropine, nausea, vomiting, shivering, and dry mouth between the two groups. Neonatal outcomes were similar in the two groups.

**Conclusion:**

Prophylactic use of glycopyrrolate does not decrease the requirements of vasopressor to prevent hypotension in non-elective cesarean section under spinal anesthesia.

**Trial registration:**

Registration number: NCT04401345. Date of registration: 26/05/2020. Website: https://clinicaltrials.gov

## Introduction

Spinal anesthesia (SA) is the commonly used anesthesia technique for cesarean section (CS). However, hypotension is frequently encountered adverse effect following spinal anesthesia [1]. Beside maternal side-effects, hypotension following spinal anesthesia may adversely affect the neonatal outcome if it is not prevented or corrected [2]. As spinal-induced hypotension may compromise the well-being of both mother and newborn, it is prudent to take preventive measures.

Based on recent meta-analysis and guidelines, the use of phenylephrine infusion with crystalloid co-loading is a reliable and recommended technique in preventing hypotension and maintaining blood pressure [3, 4]. With the use of phenylephrine, the systemic vascular resistance is restored, and cardiac output (CO) normalizes. However, the continuous infusion or larger doses of phenylephrine to prevent hypotension causes reflex bradycardia [3, 5]. Because heart rate (HR) is surrogate marker of CO, any decline in the maternal HR with the use of phenylephrine is subsequently associated with a decrease in cardiac output [5, 6].

If bradycardia associated with spinal anesthesia due to high sympathetic block occurs, then it may further worsen the hemodynamics. In addition, any fall in CO may cause fetal acidosis because uterine blood flow is dependent on maternal CO. [7] The situation is more likely to worsen during emergency cesarean sections with an already compromised fetus. Therefore, preventing any decline in maternal heart rate during CS is equally important in maintaining the CO.

In this regard, glycopyrrolate increases maternal heart rate and thereby indirectly maintains CO. [8, 9] Moreover, glycopyrrolate has minimal effect on the fetal heart rate because it does not cross placental barriers [10]. Intravenous glycopyrrolate has been investigated for its effect on hemodynamic changes after spinal anesthesia for cesarean delivery, but the results are inconsistent [8, 11, 12]. A recent meta-analysis found that prophylactic glycopyrrolate does not prevent the incidence of spinal-induced hypotension; however, it reduces the total vasopressor requirement during elective cesarean delivery under spinal anesthesia [13]. Therefore, the primary objective of this study was to find out whether the use of glycopyrrolate decreases the total amount of vasopressors to maintain hemodynamic stability during spinal anesthesia for non-elective CS.

## Methods

This was a double-blind, randomized, placebo-controlled trial conducted at the University hospital of BP Koirala Institute of Health Sciences (BPKIHS) from June 1, 2020, to January 31, 2021. This study was approved by the BPKIHS Institutional Review committee (IRC No.: IRC/1603/019) and the trial was registered prior to patient enrollment at clinicaltrials.gov (Date of registration: 26/05/2020, Registration number: NCT04401345, Principal investigator: Rajesh Deshar). The trial was conducted in accordance with the principles of the world medical association’s declaration of Helsinki (updated in 2013) and adheres to the applicable CONSORT guidelines. We enrolled consecutive term parturients of American society of Anesthesiologist (ASA) physical status (PS) II, undergoing non-elective cesarean section for category 2 and 3 [14]. Exclusion criteria included age > 40-year, body mass index > 30 kg/m^2^, height < 150 cm, maternal bradycardia (heart rate < 60/min) or tachycardia (heart rate > 100/min), hypertensive disorders, known fetal abnormalities, contraindications to spinal anesthesia or glycopyrrolate, and twin pregnancy. The written informed consent from the participant was obtained either in the labor room or in the obstetric emergency ward.

Eligible patients were randomly assigned in 1:1 ratio to glycopyrrolate (GP group) or normal saline (NS group). The anesthesia clerk (SA) generated the randomization sequence with permuted blocks of 4, 6, and 8, and stratified it according to maternal baseline heart rate (60–69 beats/min, 70–79 beats/min, 80–89 beats/min, 90–99 beats/min) using online software (https://www.sealedenvelope.com). Each study group assignment remained concealed with SA until after the patient had given consent to participate in the study. The anesthesia assistant received the concealed envelope and prepared the study medication in a sterile syringe and labeled it according to the code number after opening the envelope. Participants, investigators and attending anesthesiologists were blinded to the study groups.

Before the patient was transferred to the operating room, ranitidine 50 mg and metoclopramide 10 mg were administered intravenously via an 18 G cannula. In the operating table, patients were laid supine with a wedge placed under the right hip. Standard anesthesia monitoring including 3-lead electrocardiography, heart rate (HR), non-invasive blood pressure (NIBP), and pulse oximetry (SpO_2_) was done. A mean value of three measurements of systolic blood pressure (SBP) and HR were recorded as baseline parameters. Patency of the vein was maintained with the infusion of Ringer’s lactate solution at a minimal rate.

Before the patient was placed in a sitting position for SA, the attending anesthesiologist administered glycopyrrolate (Pyrolate®; Neon laboratories ltd., Thane, Maharashtra, India) 1 ml (0.2 mg) or NS 1 ml IV according to the randomization. After the free flow of CSF was observed, 0.5% hyperbaric bupivacaine (2.2 ml) with 10 μg fentanyl was injected intrathecally over 30 s using a 25-gauge Quincke needle at the L3–4 or L4-L5 interspace. Patients were then immediately placed supine while maintaining a 15-degree left lateral tilt. Co-loading of 1000 ml Ringer’s lactate solution was initiated at the start of spinal anesthesia using a pressure bag and it was completed within 10–15 min. Immediately after the spinal injection, phenylephrine infusion was initiated at a rate of 25 μg/min, and the infusion was titrated to maintain maternal SBP within 20% of the baseline.

The sensory level of anesthesia was determined by assessing the loss of cold sensation. Surgery was allowed once the bilateral sensory block height at T6 was achieved. Oxygen was administered via nasal cannula at 2–4 L/min until delivery. Hemodynamic parameters were recorded at the following time intervals: baseline, after the study drug was given IV, immediately after spinal anesthesia, every minute for the first 10 min, and then at 2.5 min until the end of surgery. Post-spinal hypotension, defined as SBP < 80% of the baseline reading or SBP < 100 mmHg was treated with phenylephrine 100 μg bolus and rapid infusion of Ringer’s lactate 200 ml. Infusion of phenylephrine was stopped if bradycardia (HR < 55/min) occurred without hypotension. When bradycardia (HR < 55/min) was associated with hypotension, IV ephedrine 6 mg was administered. When these measures failed to correct bradycardia, an IV atropine 0.5 mg was given. In reactive hypertension (defined as SBP > 120% of the baseline reading), the infusion of phenylephrine was stopped and restarted only when the SBP reached the target range (SBP within 120% of baseline SBP). The amount of ephedrine used was converted to phenylephrine equivalent based on the relative potency of phenylephrine to ephedrine [15]. Ondansetron 4 mg IV was administered for intraoperative nausea vomiting (IONV). Grading of intraoperative shivering was done as described previously [16] and IV meperidine 20 mg was given when the shivering involved the whole body. The primary outcome was the total amount of phenylephrine equivalent used to maintain blood pressure intraoperatively. Secondary outcomes included incidences of maternal hypotension, reactive hypertension, bradycardia, need for atropine, intraoperative nausea/vomiting, shivering, pruritus, dry mouth, dizziness; neonatal APGAR score at 1 min and 5 min, neonatal resuscitation needed, neonatal intensive care unit admission, and neonatal death.

After birth of the baby, 2 U of oxytocin was administered IV over 5–10 secs followed by an infusion of 10 U/h (oxytocin 20 U in 500 ml of Hartmann’s solution). Phenylephrine infusion was gradually tapered after delivery of the baby keeping the SBP within the target level (SBP within 120% of baseline SBP). The total amount of intraoperative IV fluids administered, and estimated blood loss were measured. Intraoperative use of other uterotonic agent or transfusion of blood was recorded. An attending pediatrician assessed neonatal Apgar scores at 1 and 5 minutes after delivery.

### Statistical analysis

Normality of data was checked using histogram, Kurtosis Skewness test, and Shapiro-Wilk test. For continuous variables with normal and non-normal distribution, mean (SD) and median (interquartile range) were used respectively. For categorical variables, the percentage of frequency was used. Student t-test and Mann–Whitney U-test were applied for continuous data which showed normal and non-normal distribution respectively. The categorical data was compared using the chi-square test. Fisher exact test was used instead, when the expected values in any of the cells of a contingency table were < 5. A *P* value of less than 0.05 was considered statistically significant. All analyses were conducted using STATA version 15.0 (Stata Corporation, College Station, TX, USA).

Sample size calculation was based on the mean amount of phenylephrine required for maintaining maternal hemodynamics during elective cesarean section under spinal anesthesia which was 501 (154) μg in parturients receiving glycopyrrolate as compared to 552 (118) μg in those who did not receive glycopyrrolate [9]. To detect this difference, we needed 114 subjects in each group with a power of 80%, at a two-sided alpha level of 0.05. Allowing for a 15% dropout rate during the study period, a total of 258 patients were enrolled (STATA version 15.0, Stata Corporation, College Station, TX, USA).

## Results

**Fig. 1:**
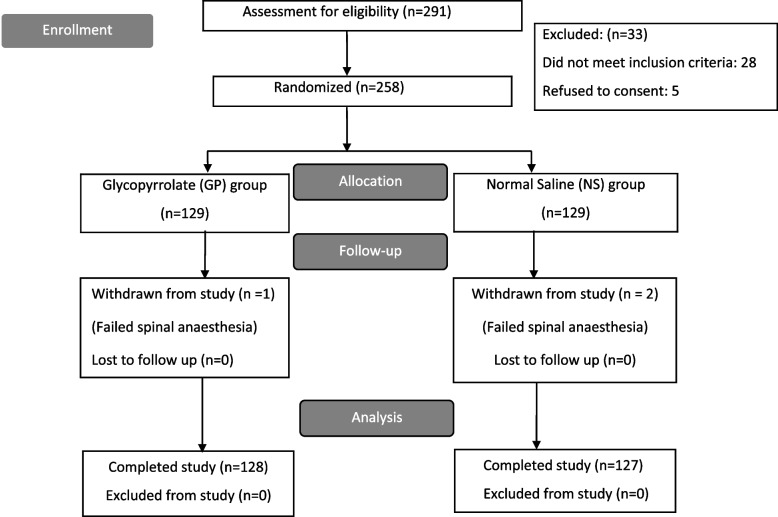
Patient enrollment, randomization, and analysis

A total of 291 parturients of ASA PS II category 2 and 3 planned for CS under SA were assessed for the eligibility. 258 patients were enrolled in the study as 28 patients did not meet the inclusion criteria, and five did not give consent. After randomization, one patient in the glycopyrrolate group and two patients in the saline group had failed spinal anesthesia. 255 patients completed the study, 128 patients in glycopyrrolate and 127 patients in placebo group (Fig. 1).

Both the glycopyrrolate and normal saline groups were comparable with respect to baseline characteristics (Table 1). No statistically significant difference was noted between the two groups regarding surgical and spinal block profiles, total fluid received, and blood loss during surgery (Table 2).

**Table 1:** Baseline characteristics of the study participants

Variables	Glycopyrrolate Group (***n*** = 128)	Saline Group (***n*** = 127)	***P***
Age (years)	25.92 ± 4.93	26.10 ± 4.63	0.77
Height (cm)	154 (152–156)	153 (151–156)	0.14
Weight (kg)	61.72 ± 7.17	61.27 ± 6.67	0.60
Body mass index (kg/m^2^)	25.85 ± 2.59	25.88 ± 2.65	0.94
Period of gestation (weeks)	40 (38–40)	39 (38–40)	0.08
Indication for cesarean section			0.39
Failed induction	29 (23)	23 (18)	
Meconium-stained liquor	20 (16)	19 (15)	
Previous cesarean section	28 (22)	43 (34)	
Oligohydramnios	12 (9)	9 (7)	
Abnormal lie	13 (10)	9 (7)	
Nonreactive non-stress test	17 (13)	19 (15)	
Cephalo-pelvic disproportion	9 (7)	5 (4)	
Systolic blood pressure (mmHg)	123.59 ± 11.87	122.53 ± 9.07	0.42
Heart rate (beats/min)			0.93
60–69	9 (7)	10 (8)	
70–79	25 (19)	21 (17)	
80–89	38 (30)	40 (31)	
90–99	56 (44)	56 (44)	

Values are in mean ± SD, median (interquartile range), number (percentage)

**Table 2:** Surgical profiles and spinal block height characteristics

Variables	Glycopyrrolate Group (***n*** = 128)	Saline Group (***n*** = 127)	***P***
Induction to skin incision (min)	6 (5–8)	6 (5–8)	0.91
Induction to delivery time (min)	12.75 (10–15)	12 (10–15)	0.76
Uterine incision to delivery time (sec)	50 (35–60)	50 (40–60)	0.22
Maximum spread of block height (thoracic dermatome, T)	4 (4–4)	4 (4–4)	0.49
Time to reach block height T6 (min)	3 (2–4)	2 (2–4)	0.18
Total duration of surgery (min)	49.73 ± 11.55	48.68 ± 10.54	0.45
Fluid received (ml)	1550.94 ± 244.92	1574.02 ± 240.27	0.45
Blood loss (ml)	562.27 ± 119.70	580.24 ± 128.63	0.24

Values are in mean ± SD, median (interquartile range)

**Fig. 2:**
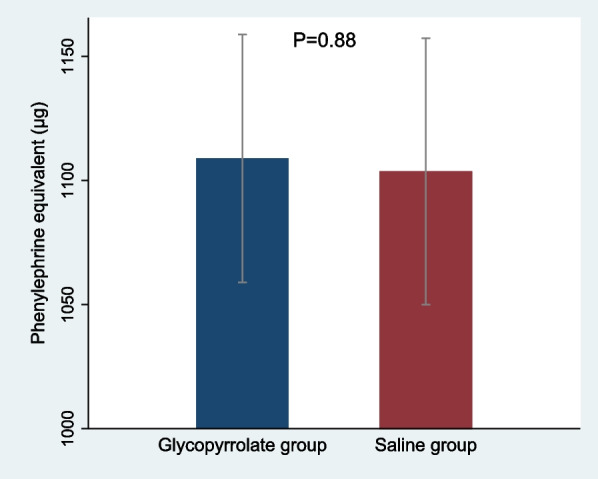
Intraoperative mean phenylephrine equivalent consumed in glycopyrrolate and saline group. Error bar indicating 95% confidence interval

The mean phenylephrine equivalent needed for maintaining intraoperative maternal hemodynamics was 1108.96 μg in the glycopyrrolate group and 1103.64 μg in the saline group, and the difference between groups was 5 μg (95% confidence interval [CI], − 67.97 to 78.62; Fig. 2). The overall incidence of hypotension was 30% in the glycopyrrolate group and 39% in the saline group (*P* = 0.13) (Table 3). There was no statistically significant difference in recurrence of hypotensive episodes and bradycardia between the glycopyrrolate and saline groups (Table 3). The mean (SD) lowest SBP was 99.78 (11.28) mmHg in the glycopyrrolate group, as compared to 95.21 (11.63) mmHg in the saline group (mean difference, 4.57; 95% CI, 1.7 to 7.4; *P* = 0.001). Tachycardia (HR > 100 beats/min) was reported in 70% of the participants in the glycopyrrolate group, and in 57% of those in the saline group (*P* = 0.04) (Table 3).

**Table 3:** Intraoperative maternal hemodynamics, adverse effects, and use of uterotonic agent

Variables	Glycopyrrolate group (***n*** = 128)	Saline group (***n*** = 127)	***P***
Overall hypotension	38 (30)	49 (39)	0.13
Episodes of hypotension (> 1)	20 (16)	28 (22)	0.20
Lowest SBP (mmHg)	99.79 ± 11.29	95.21 ± 11.64	0.001
Bradycardia (HR < 55/min)	3 (2)	7 (6)	0.22
Maternal tachycardia (HR > 100/min)	89 (70)	73 (57)	0.04
Reactive hypertension (SBP > 20% baseline)	13 (10)	7 (6)	0.17
Nausea	18 (14)	23 (18)	0.39
Vomiting	9 (7)	15 (12)	0.20
Shivering	13 (10)	18 (14)	0.35
Dry mouth	66 (52)	51 (40)	0.08
Atropine used for bradycardia	1 (1)	3 (2)	0.31
Pethidine used for shivering	6 (5)	12 (9)	0.22
Oxytocin used (Units)	6.84 ± 2.37	7.18 ± 2.92	0.49
Carboprost	17 (13)	22 (17)	0.24
Methylergonovine	6 (5)	3 (2)	1.00
Carboprost + methylergonovine	8 (6)	11 (9)	0.49

Values are in mean ± SD, number (percentage)

The incidences of other maternal adverse events (reactive hypertension, nausea, vomiting, shivering, dry mouth) were comparable between the glycopyrrolate and saline groups (Table 3). None of the participants reported dizziness in either group. Neonatal outcomes are depicted in Table 4. There were no neonatal deaths.

**Table 4:** Comparison of neonatal outcomes between two groups

Neonatal Outcome	Glycopyrrolate Group (***n*** = 128)	Saline Group (***n*** = 127)	***P***
Apgar score at 1 min	8 (7–8)	8 (7–8)	0.65
Apgar score at 5 min	9 (8–9)	9 (8–9)	0.35
Apgar score < 7 at 1 min	9 (7)	11 (14)	0.27
Apgar score < 7 at 5 min	2 (2)	2 (2)	0.99
Resuscitation required	2 (2)	4 (3)	0.45
NICU admission	2 (2)	3 (2)	0.68

Values are in median (interquartile range), number (percentage). *Abbreviations*: *NICU* Neonatal intensive care unit

## Discussion

We found that prophylactic use of IV glycopyrrolate in the non-elective cesarean section did not show any significant difference in the vasopressor requirement compared to the normal saline group. Similarly, use of the IV glycopyrrolate did not result in a significant difference in the incidence of post-spinal hypotension. No difference was detected in terms of IONV, shivering, or dry mouth between the two groups.

A meta-analysis showed a modest decrease in phenylephrine equivalent requirement with glycopyrrolate compared to control [13]. The studies included in the meta-analysis had reported the vasopressor requirement as a secondary outcome measure [9, 11, 12, 17]. One of the reasons behind the contrary result in our study may be that the previous studies were not powered enough to detect the differences in the vasopressor requirement. Second, there is variation in the dose and/or timing of glycopyrrolate administration, and this may influence the outcomes measures. Yoon HJ and colleagues administered glycopyrrolate IV 0.2 mg immediately after spinal anesthesia for the elective cesarean section [9]. They reported a significant difference in total phenylephrine requirement. In their study phenylephrine was infused at a rate of 50 μg/min for 15 min whereas in our study phenylephrine infusion was initiated at a rate of 25 μg/min and we continued it till the end of surgery. The continuous fixed infusion of phenylephrine may have masked the blood pressure fluctuation. As a result, no difference was detected in the total phenylephrine equivalent requirement. Finally, all previous studies were conducted in elective cesarean sections and therefore, the similar outcome cannot be extrapolated in emergency cesarean section due to differences in hemodynamics.

The meta-analysis revealed no statistically significant reduction in hypotension when the prophylactic glycopyrrolate was compared with the placebo [13]. Among the four studies included in the meta-analysis, a wide variation in the administration of the vasopressors was apparent. Two studies had used prophylactic phenylephrine, while in the other two studies, the authors did not use prophylactic vasopressor to prevent post-spinal hypotension. Nevertheless, whether the prophylactic vasopressor was used or not, pretreatment with glycopyrrolate did not offer any protection against post-spinal hypotension. We too observed no significant reduction in post-spinal hypotension when a combination of glycopyrrolate and phenylephrine was used prophylactically against phenylephrine alone. Interestingly, we did find a significant difference in the lowest systolic blood pressure (SBP) recorded. The mean lowest SBP recorded in the glycopyrrolate was 99.79 ± 11.29 mmHg compared to 95.21 ± 11.64 mmHg in the placebo group (*P* = 0.001). In our study, one of the criteria for defining post-spinal hypotension was SBP < 100 mmHg. In both the groups the mean lowest SBP recorded was < 100 mmHg. As a result, the incidence of post-spinal hypotension was comparable between the two groups.

In our study, the maternal heart rate was significantly higher in the glycopyrrolate group. Glycopyrrolate being an anticholinergic has a direct effect on the heart to increase its rate [18]. Results from the meta-analysis showed that the administration of glycopyrrolate significantly increased the heart rate [13]. Phenylephrine and ephedrine are two commonly used vasopressors as prophylactic or treatment measures to counter post-spinal hypotension. Ephedrine through its cardiac β-adrenoceptor agonist effects causes tachycardia [19] while phenylephrine is associated with decreased maternal heart rate [5]. Therefore, logically the effect on the heart rate may be influenced by the choice of vasopressor. However, when prophylactic glycopyrrolate and phenylephrine were used in combination, the maternal heart rate was higher than when phenylephrine was used alone. In our study, although 7 (6%) patients in the phenylephrine group had bradycardia (HR < 55/min) compared to 3 (2%) patients in the glycopyrrolate and phenylephrine combination group, the difference was not statistically significant. As a result, pretreatment with glycopyrrolate may not offer protection against phenylephrine-induced reflex bradycardia after spinal anesthesia for cesarean delivery.

A previous study reported that episodes of reactive hypertension were higher in the glycopyrrolate and phenylephrine group (20 [44%] versus 8 [16%] in the phenylephrine group alone, *P* = 0.007) [8]. In the above study, the investigators had used the phenylephrine infusion at 50 μg/min. In contrast, we used the infusion of phenylephrine at 25 μg/min. Phenylephrine-induced reactive hypertension is dose-dependent, with higher episodes of reactive hypertension associated with increasing doses [20]. This may be a reason why no significant difference was noted in the episodes of reactive hypertension in our study.

Previous studies have shown conflicting results in terms of IONV [8, 11, 12, 21]. Two studies have shown that prophylactic administration of glycopyrrolate reduced the incidence of nausea during spinal anesthesia for cesarean delivery [11, 21]. In those studies, prophylactic vasopressors were not used for preventing post-spinal hypotension. Whereas when prophylactic vasopressors were used, pretreatment with glycopyrrolate did not offer any advantage in terms of IONV [8, 12]. In current practice, prophylactic administration of vasopressors especially phenylephrine is recommended for cesarean delivery under neuraxial anesthesia. This strategy not only reduces the incidence and severity of post-spinal hypotension but also prevents the occurrence of IONV. Therefore, prophylactic use of glycopyrrolate may not be helpful in reducing the episodes of IONV when phenylephrine is co-administered. However, IONV during spinal anesthesia for CS has multiple contributing factors, which include hypotension, vagal hyperactivity, visceral pain, rescue IV opioid, uterotonic agents [22]. Since IONV is complex in nature; it is difficult to establish the causal relationship between prophylactic glycopyrrolate and its effect on IONV. Further study on the direct effect of glycopyrrolate on nausea and vomiting is needed to reach the conclusion.

Glycopyrrolate has antisialagogue property and dry mouth is a common side-effect. Previous studies have reported a higher incidence of dry mouth in the glycopyrrolate group compared with the control group [8, 12]. Of the five studies included in the meta-analysis, two reported the effect of prophylactic glycopyrrolate on the incidence of dry mouth with a relative risk of 5.5 [99% CI, 1.82–14.57] in comparison to placebo [13]. In our study, although more number of parturients who received glycopyrrolate complained of dry mouth, the difference was not statistically significant.

## Conclusion

The prophylactic use of the glycopyrrolate does not decrease the requirement of vasopressors to prevent hypotension in the non-elective cesarean section under spinal anesthesia.

## Data Availability

The datasets used and/or analysed during the current study are available from the corresponding author on reasonable request.

## Abbreviations

SA: Spinal Anesthesia
CS: Cesarean section
CO: Cardiac output
IRC: Institutional review committee
CONSORT: Consolidated Standards of Reporting Trials
ASA PS: American Society of Anesthesiology Physical Status
BMI: Body mass index
HR: Heart rate
NIBP: Non-invasive blood pressure
SpO_2_: Pulse oximetry
SBP: Systolic blood pressure
IV: Intravenous
CSF: Cerebrospinal fluid
NICU: Neonatal intensive care unit
IONV: Intraoperative nausea vomiting
SD: Standard deviation
